# Binding Mechanism of Inhibitors to BRD4 and BRD9 Decoded by Multiple Independent Molecular Dynamics Simulations and Deep Learning

**DOI:** 10.3390/molecules29081857

**Published:** 2024-04-19

**Authors:** Jian Wang, Wanchun Yang, Lu Zhao, Benzheng Wei, Jianzhong Chen

**Affiliations:** 1 School of Science, Shandong Jiaotong University, Jinan 250357, China; 2 Center for Medical Artificial Intelligence, Shandong University of Traditional Chinese Medicine, Qingdao 266112, China

**Keywords:** BRD4, BRD9, molecular dynamics simulations, deep learning, binding free energy

## Abstract

Bromodomain 4 and 9 (BRD4 and BRD9) have been regarded as important targets of drug designs in regard to the treatment of multiple diseases. In our current study, molecular dynamics (MD) simulations, deep learning (DL) and binding free energy calculations are integrated to probe the binding modes of three inhibitors (H1B, JQ1 and TVU) to BRD4 and BRD9. The MD trajectory-based DL successfully identify significant functional function domains, such as BC-loop and ZA-loop. The information from the post-processing analysis of MD simulations indicates that inhibitor binding highly influences the structural flexibility and dynamic behavior of BRD4 and BRD9. The results of the MM-GBSA calculations not only suggest that the binding ability of H1B, JQ1 and TVU to BRD9 are stronger than to BRD4, but they also verify that van der Walls interactions are the primary forces responsible for inhibitor binding. The hot spots of BRD4 and BRD9 revealed by residue-based free energy estimation provide target sites of drug design in regard to BRD4 and BRD9. This work is anticipated to provide useful theoretical aids for the development of selective inhibitors over BRD family members.

## 1. Introduction

Bromodomains (BRDs) are evolutionarily conserved epigenetic reader modules that play functional roles in identifying N-acetylated lysine (KAc) residues on histones and other proteins [1,2]. Histone tails undergo various post-translational modifications that participate in the regulation of chromatin accessibility [3,4]. The recognition motifs of BRDs on KAc residues cooperate with other chromatin factors to control gene transcription regulation [5]. Currently, BRDs are divided into eight families, consisting of at least 56 nuclear or cytoplasmic proteins in humans, based on their diverse structures and functions [1,6,7,8]. Previous studies have shown that BRD proteins, particularly those of the bromodomain and extraterminal (BET) family, are involved in tumorigenesis, cancers and inflammatory diseases. Moreover, there is growing evidence to suggest that BRDs play a role in the development of various diseases by regulating the transcription of several genes involved in cancer growth and inflammation [9,10,11]; therefore, BRD proteins have been an important target in regard to anti-cancer drug design. In addition, different BRD inhibitors have been designed, some of which are even undergoing clinical trials for both oncology and non-oncology indications [12,13,14,15]. Therefore, insights into the binding mechanism of inhibitors to BRDs are essential for drug development targeting BRDs.

**Figure 1:**
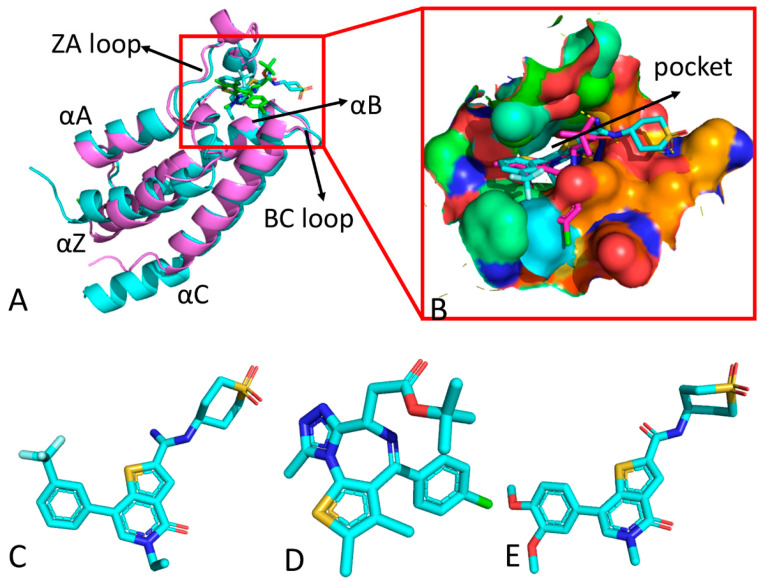
Molecular structures: (**A**) structural superimposition of inhibitor-bound BRD4/BRD9 complexes, (**B**) binding pocket of two BRD proteins, (**C**) H1B, (**D**) JQ1 and (**E**) TUV. In this figure, BRD4 and BRD9 are shown in cartoon modes and inhibitors are displayed in stick modes.

In spite of large sequence variations, all BRD proteins share a common topology structure with a left-handed bundle of four alpha helices (αZ, αA, αB and αC) connected by functional loop regions (ZA and BC loops) that are responsible for substrate specificity (Figure 1A), and their binding pocket is depicted in Figure 1B. Among the family members of BRDs, BRD4 is one of the most widely studied proteins [14,16,17,18,19], as it binds to transcriptional start sites of genes expressed during the M/G1 transition, which affects mitotic progression [20]. Based on functional studies, abnormalities in BRD4 can result in disorders of gene expression, which play a key role in the occurrence and development process of tumors [10,21,22,23]. Disturbance of BRD4 activity mediated by inhibitors contributes potential therapeutic benefits in regard to cancer, inflammation, and immunology. However, mechanism information on inhibitor-mediated disturbances in the activity of BRD4 is still insufficient. Thus, it is of high significance to probe the atomic-level binding mechanisms of inhibitors to BRD4 for the development of highly secure small molecule inhibitors targeting BRD4.

As a member of the BRD family, BRD9 has received special attention as a druggable subunit of mSWI/SNF chromatin-remodeling complexes. Mutations from various subunits of these complexes have been found in nearly 20% of all human cancers [4,24,25,26]. Karim et al. investigated inhibitor selectivity in the BRD7/9 subfamily, and their results provided a new framework for the development of BRD7/9 inhibitors with improved selectivity or additional polypharmacologic properties [24]. Theodoulou et al. reported the first selective cellular chemical probe (I-BRD9) for BRD9, and this probe can be used for recognizing genes regulated by BRD9 in Kasumi-1 cells, which are involved in oncology and immune response pathways [4]. To date, small-molecule inhibitors targeting KAc sites have been designed for the mSWI/SNF complexes in cancer [27,28,29,30,31]. Despite these advances, the mechanism information regarding the binding of inhibitors to BRD9 is currently limited. Therefore, further exploration of the binding modes of inhibitors to BRD9 is highly significant in terms of the development of clinically available anti-cancer drugs targeting BRDs.

Recently, there has been a focus on anti-cancer drugs regarding insights into the binding selectivity of inhibitors towards proteins of the BRD family [13,31,32,33,34,35,36]. An inhibitor called LP99, reported by Clark et al., has shown a preference for inhibiting the association of BRD7 and BRD9 with acetylated histones in vitro and in cells [27]. Two inhibitors, BT0 and BS6, proposed by Jiang et al., have a selectivity of more than 100-fold for BRD4(1) over BRD4(2). Moreover, their work has indicated that selectively modulating individual BRDs could be a strategy for the treatment of acute gouty arthritis [37]. Su et al. utilized molecular dynamics (MD) simulations and binding free energy calculations to investigate the binding selectivity of inhibitors to BRD9 and BRD4. Their results clarified that two highly flexible loops, the ZA-loop and BC-loop, significantly contribute to the binding selectivity of inhibitors to BRD9 and BRD4 [38]. Wang et al. conducted multiple short MD simulations to study the binding selectivity of inhibitors to BRD9 and TAF1(2). Their work revealed that enthalpy contributions play a critical role in the selective recognition of inhibitors towards BRD9 and TAF1(2) [39]. Considering the important roles of BRD4 and BRD9 in diseases, it is necessary to explore the molecular mechanisms underlying the binding selectivity of inhibitors to BRD4 and BRD9 in order to develop highly selective inhibitors targeting BRDs.

Among the various computational tools used for investigating inhibitor−protein binding, MD simulations [40,41,42,43,44,45,46], Gaussian accelerated molecular dynamics (GaMD) simulations [47,48,49] and binding free energy predictions [50,51,52,53,54] have been popular methods. Furthermore, conventional MD (cMD) and GaMD simulations were applied to successfully explore inhibitor-induced conformational changes [55,56,57,58,59]. Recently, MD simulations have shown a power in applications in regard to discovering cysteine protease inhibitors and molecular-level information on drug design [60]. To extract essential information about protein conformational dynamics from trajectories, both cMD and GaMD simulations have been combined with machine learning and deep learning (DL). Plante et al. utilized MD simulations and machine learning to successfully identify the molecular determinants involved in the classification of G protein-coupled receptor (GPCR) structures and functions [61,62]. Miao’s group developed a method called GaMD, involving deep learning (DL) and a free energy profiling workflow (GLOW), to gain further mechanistic insights into GPCR function, and their results align well with previous experimental and computational studies [63,64]. We will adopt a cMD trajectory-based DL to enhance our understanding of the binding selectivity of inhibitors towards BRD4 and BRD9.

For this study, three inhibitors, namely JQ1, H1B and TVU, were selected to examine their binding selectivity towards BRD4 and BRD9. The structures of the three inhibitors are illustrated in Figure 1C–E. JQ1 is a novel thieno-triazolo-1,4-diazepine compound that possesses a bulky t-butyl ester functional group at C6. Additionally, binding of (+)-JQ1 significantly enhances the thermal stability of all BET family BRDs [36]. H1B, also known as I-BRD9, exhibits more than 700-fold selectivity towards the BET family and over 200-fold selectivity over the highly homologous BRD7 [4,24]. The IC50 value of the TVU against BRD9 is 10 nM, indicating its stronger inhibitory activity on BRD9 [4]. Therefore, gaining insights into the binding selectivity of these three inhibitors towards BRD4 and BRD9 is crucial for designing highly selective inhibitors targeting the BRD family. To achieve our objective, multiple independent MD (MIMD) simulations, DL, principal component analysis (PCA) [65,66,67,68,69] and binding free energy prediction were integrated in this study to decipher the binding selectivity of inhibitors in regard to BRD4 over BRD9. It is anticipated that this study will provide valuable theoretical guidance for the development of drugs targeting the BRD family.

## 2. Results and Discussion

### 2.1. Difference in Contacts of Structural Domains Revealed by Deep Learning

**Figure 2:**
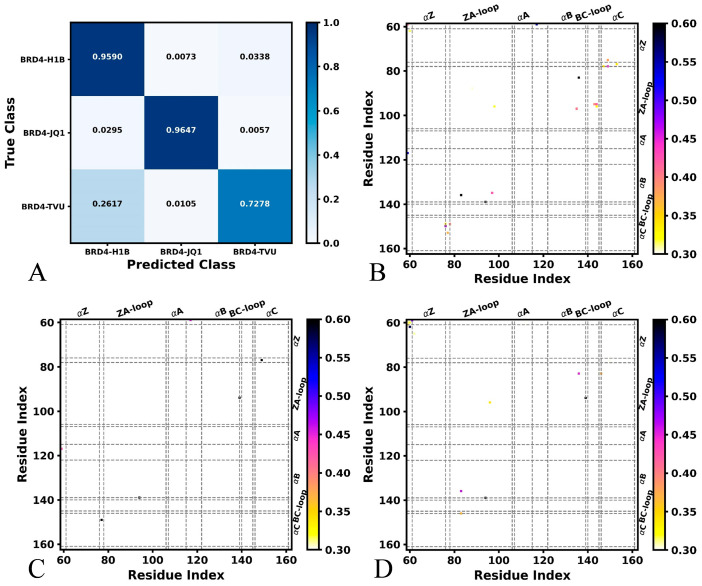
Salience maps of key residues detected by DL from MD trajectory of inhibitor-bound BRD4: (**A**) classification of different BRD4 systems bound by H1B, JQ1 and TVU, (**B**) salience map of H1B-bound BRD4, (**C**) salience map of JQ1-bound BRD4 and (**D**) salience map of TVU-bound BRD4.

**Figure 3:**
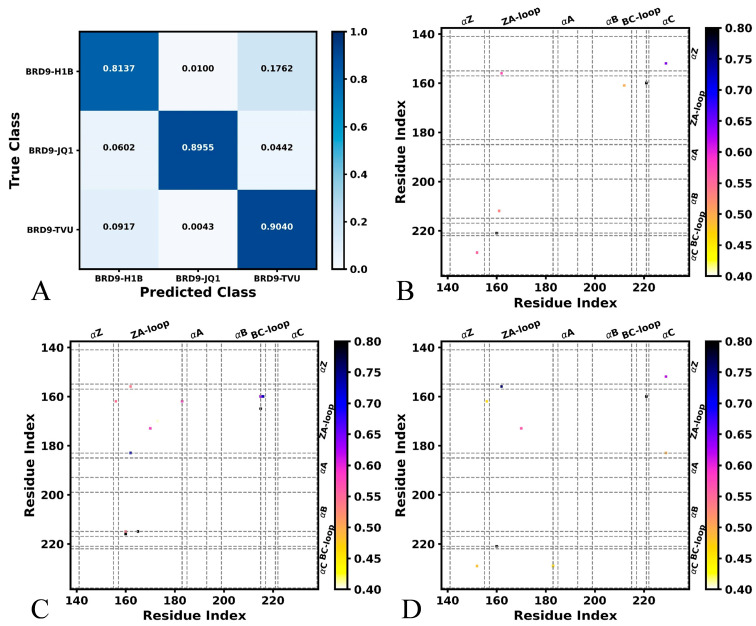
Salience maps of key residues detected by DL from the MD trajectory of inhibitor-bound BRD9: (**A**) classification of different BRD9 systems bound by H1B, JQ1 and TVU, (**B**) salience map of H1B-bound BRD9, (**C**) salience map of JQ1-bound BRD9 and (**D**) salience map of TVU-bound BRD9.

The process of the DL is described in Figure S1. Firstly, the conformations recorded in the MD trajectories are transformed into the images used for the DL. Secondly, the images are randomly divided into a training set and a validation set to perform the DL. Lastly, the trained results are visualized in Figure 2, Figure 3 and Figure S2. The details for the DL are clarified in the later method section. The inhibitor-bound BRD4 and BRD9, respectively, performed classification through DL on images extracted from the SMT using the MDTraj program. For the inhibitor-bound BRD4 (Supporting Information Figure S2A,B), the overall accuracy achieves 88.47% on the validation set after 30 epochs, while the overall loss reaches 0.281. Among 4000 snapshots for the validation of each system, three systems can be basically accurately identified (Figure 2A). The 3836 snapshots of H1B-BRD4, the 3859 snapshots of JQ1-BRD4 and the 2911 snapshots of TVU-BRD4 are accurately recognized. The 29 and 135 snapshots of H1B-BRD4 are inaccurately identified as JQ1-BRD4 and TVU-BRD4, respectively. From 4000 snapshots of the validation set of JQ1-BRD4, the 118 snapshots inaccurately recognized as H1B-BRD4 and 23 are inaccurately identified as TVU-BRD4. The 1047 and 42 snapshots from 4000 TVU-BRD4 are erroneously recognized as H1B-BRD4 and JQ1-BRD4, individually. The pixel-attributed residue contact gradient maps of the most populated BRD4 structures are bounded by H1B, JQ1 and TVU (Figure 2B–D), respectively. On the whole, the characteristic residue contacts of H1B-bound BRD4 are located between helixes αB and ZA-loop, as well as αC and ZA-loop (Figure 2B). The characteristic residue contacts of JQ1-bound BRD4 are situated between αC and αZ as well as αB and the ZA-loop (Figure 2C). Compared to the H1B-bound BRD4, binding of JQ1 leads to the appearance of contacts between αC and the ZA-loop. The characteristic residue contacts of TVU-bound BRD4 occur between αC and αZ, αA and αZ, αC and the ZA-loop, as well as αB and the ZA-loop (Figure 2D). By comparison with the H1B-bound BRD4, binding of TVU induces the appearance of two new contacts of αZ with αC and αA. The previous experimental work indicated that the ZA-loop, BC-loop, αC and αZ play important roles in the binding of inhibitors to BRD4 [17,36], which supports our current findings.

With respect to the inhibitor-bound BRD9, the overall accuracy achieves 87.20% on the validation set after 30 epochs, while the overall loss reaches 0.259 (Figure S2C,D). Among 4000 snapshots for the validation of each system, three systems can be accurately identified (Figure 3A). In detail, the 3255 frames of H1B-BRD9, 3582 frames of JQ1-BRD9 and 3616 frames of TVU-BRD9 are accurately identified (Figure 3A). The 40 and 705 frames of H1B-BRD9 are inaccurately recognized as JQ1-BRD9 and TVU-BRD9, separately. Among the 4000 frames of the validation set of JQ1-BRD9, 241 frames are inaccurately identified as H1B-BRD9 and 177 are inaccurately identified as TVU-BRD9. The 367 and 17 snapshots from 4000 TVU-BRD9 are erroneously recognized as H1B-BRD9 and JQ1-BRD9, respectively. The pixel-attributed residue contact gradient maps of the most populated BRD9 structures are associated by H1B, JQ1 and TVU (Figure 3B–D), respectively. In total, the characteristic residue contacts of H1B-bound BRD9 are situated between helixes αB and ZA-loop, as well as αC and αZ (Figure 3B). The characteristic residue contacts of JQ1-bound BRD9 are located between αZ and the ZA-loop, as well as the ZA-loop and BC-loop (Figure 3C). Compared to the H1B-bound BRD9, binding of JQ1 induces the appearance of contacts between αZ and the ZA-loop as well as the ZA-loop and BC-loop, but it leads to the disappearance of contacts between αB and the ZA-loop as well as αC and αZ (Figure 3A,D). The characteristic residue contacts of TVU-bound BRD9 primarily occur between αC and αZ, the BC-loop and ZA-loop, αC and the ZA-loop and αC and the BC-loop (Figure 3D). By comparison with the H1B-bound BRD9, the presence of TVU induces the appearance of the contacts between the BC-loop and ZA-loop, αC and the ZA-loop and αC and the BC-loop, but binding of TVU also leads to the disappearance of the contacts of αC with the ZA-loop and αB (Figure 3B,D). In a previous experimental study, it was observed that the ZA-loop, BC-loop, αC and αZ play important roles in the binding of inhibitors to BRD9, which is in basic agreement with our results [4,70].

According to the above analyses, the trajectory-based DL can rationally identify significant structure domains of BRD4 and BRD9, which is involved in the binding of inhibitors. Meanwhile, the DL also captures the difference between contacts of structural domains between BRD9 and BRD4. Compared to the inhibitor-bound BRD4, more structural contacts were found in the inhibitor-bound BRD9, which may imply the functional difference between BRD4 and BRD9.

### 2.2. Free Energy Profiles and Structural Dynamics of BRD4 and BRD9

To understand the dynamics behavior of BRD4 and BRD9, PCA was performed on the inhibitor-bound BRD4 and BRD9 by using the coordinates of the Cα atoms recorded at an MD trajectory. The function of eigenvalues as eigenvector indexes was depicted in Figure S3. The eigenvalues are usually used to describe the structural fluctuation of proteins along the first eigenvectors. The first six eigenvalues account for 69.15, 79.58 and 69.87% of the total movements for the H1B-, JQ1- and TVU-bound BRD4 (Figure S3A), respectively, while the first six eigenvalues account for 77.89, 75.99 and 73.49% of the total movements of the H1B-, JQ1- and TVU-bound BRD9 (Figure S3B), individually. On the whole, the first eigenvalues of the inhibitor-bound BRD9 are greater than that of the inhibitor-bound BRD4, indicating that the fluctuation amplitude of BRD9 along the first eigenvector is stronger than that of BRD4.

**Figure 4:**
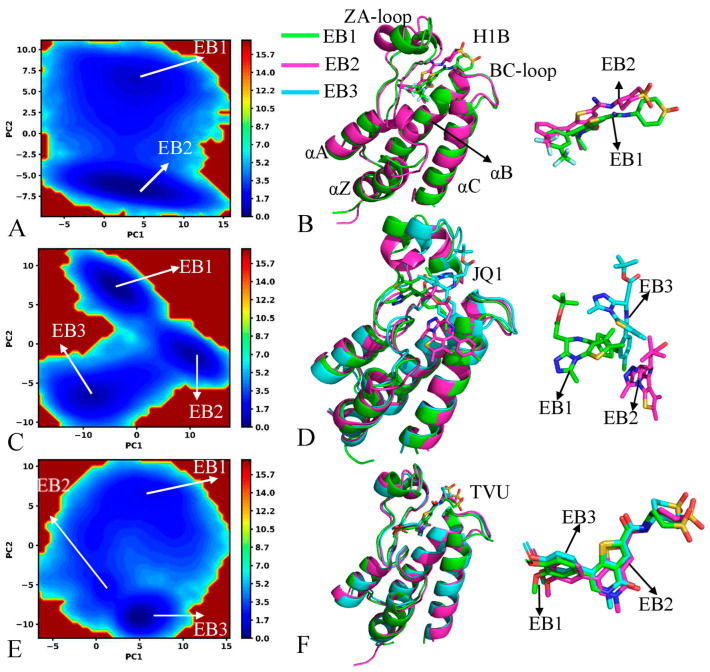
Free energy profiles and representative structures of the inhibitor-bound BRD4: (**A**,**C**,**E**) corresponding to FELs of the H1B-, JQ1- and TVU-bound BRD4, respectively, and (**B**,**D**,**F**) indicating superimposition of representative structures for BRD4 and inhibitors trapped in different EBs. The free energy is scaled in kcal/mol and BRD4 is shown in cartoon modes.

To probe the difference in free energy profiles of BRD4 and BRD9, the projections of MD trajectories onto the first two eigenvectors were used as reaction coordinates to build free energy landscapes (FELs), with the results being depicted in Figure 4 and Figure S4. MD simulations identify two, three and three energy basins (EBs) in the H1B-, JQ1- and TVU-bound BRD4 (Figure 4A,C,E), respectively, in which JQ1 produces the most significant effect on the free energy profiles of BRD4. By structural comparison of H1B, JQ1 and TVU, it is found that JQ1 brings a net positive charge, which may produce strong electrostatic interactions with those charged residues in BRD4. To check the structural difference in different EBs, the representative structures falling into the EBs were superimposed together (Figure 4B,D,F). The ZA-loop of BRD4 produces the mostly evident difference among different energy states, in particular the JQ1-bound BRD4 (Figure 4D). Meanwhile, two inhibitors, H1B and TVU, hardly yield big deviations in the binding pocket of BRD4 (Figure 4B,F), and only H1B slides slightly. In contrast to H1B and TVU, JQ1 generates large deviations among three representative structures and has three different binding poses.

On the other hand, these results indicate that strong electrostatic interactions caused by a net positive charge of JQ1 produce an obvious effect on the binding poses of JQ1 in the binding pockets of BRD4 and BRD9. For the BRD9, MD simulations detect three, four and two EBs in the H1B-, JQ1- and TVU-bound BRD9 (Figure S4A,C,E) individually. Similar to BRD4, JQ1 also produces the most significant influences on free energy profiles of BRD9. The representative structures trapped in different EBs were aligned together to examine structural differences among different energy states (Figure S4B,D,F). The ZA-loop and BC-loop of BRD9 generate evident deviations between different EBs; moreover, the structural deviations of the ZA-loop are much higher than the BC-loop, and the ZA-loop shows highly disordered states. Three inhibitors, H1B, JQ1 and TVU, yield obvious deviations in the binding pocket of BRD9, in particular JQ1. By comparison, the structural stability of H1B, JQ1 and TVU in the binding pocket of BRD9 is weaker than in BRD4, which may exert significant impacts on inhibitor binding.

In order to probe the dynamics behavior of BRD4 and BRD9, the first eigenvector was visualized and the results were depicted in Figure S5. It is observed that binding of three inhibitors brings about a different effect on the conformations of BRD4 and BRD9, but the common structural domains of BRD4 and BRD9 affected by inhibitor binding are primarily located at the ZA-loop and BC-loop. Compared to H1B and TVU, binding of JQ1 highly strengthens the structural fluctuations of the ZA-loop from BRD4 and BRD9 along the first eigenvector (Figure S5B,E). On the other hand, binding of three inhibitors enhances the structural fluctuation of the ZA-loop in BRD9 by comparison with that of the BRD4 (Figure S5). In addition, binding of JQ1 to BRD9 also strengthens the structural fluctuation of the helix αA relative to binding of JQ1 to BRD4 (Figure S5B,E). Based on the aforementioned analyses, the electrostatic property of JQ1 yields more obvious impacts on conformations of BRD4 and BRD9, especially BRD4.

Based on the current PCA, the binding of H1B, JQ1 and TVU produces a different effect on the dynamics behavior of BRD4 and BRD9, in particular JQ1, indicating that (1) the fluctuation amplitude of BRD9 bounded by three inhibitors along the first eigenvector is stronger than that of BRD4, (2) the structural stability of three current inhibitors in the binding pocket of BRD9 is weaker than in BRD4 and (C) that the presence of three inhibitors strengthens the structural fluctuation of the ZA-loop in BRD9 relative to BRD4. These results indicate that electrostatic properties should be given special attention in future designs of selective inhibitors in regard to the BRD family.

### 2.3. Structural Property of BRD4 and BRD9

**Figure 5:**
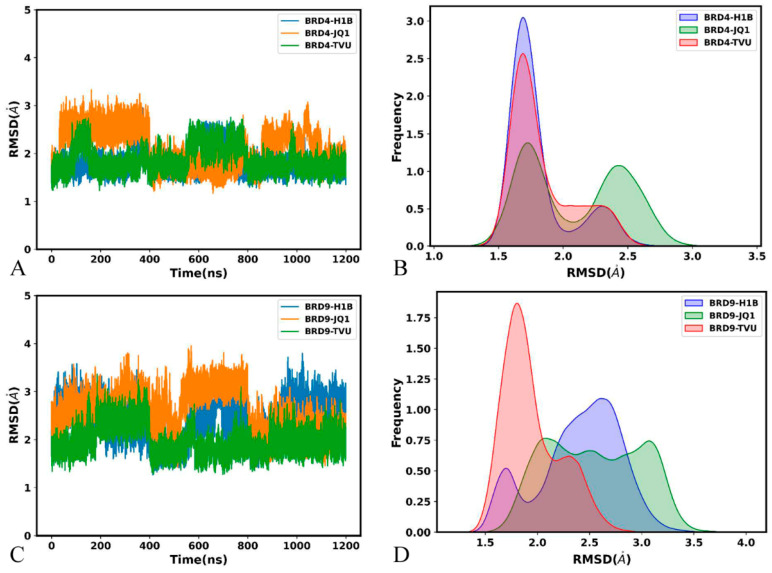
RMSDs of backbone atoms in BRD4 and BRD9: (**A**) the time course of RMSDs for BRD4, (**B**) the frequency distribution of RMSDs for BRD4, (**C**) the time course of RMSDs for BRD9 and (**D**) the frequency distribution of RMSDs for BRD9.

In order to uncover the structural fluctuation, the root-mean-square deviations (RMSDs) of backbone atoms from BRD4 and BRD9 were calculated through the entire MD simulations by referencing the initially optimized structures (Figure 5). The RMSDs of the inhibitor-bound BRD4 fluctuate from 1.17 to 3.33 Å (Figure 5A), while those of the inhibitor-bound BRD9 are located at a range of 1.26–3.96 Å (Figure 5C), indicating that the inhibitor-bound BRD9 shows bigger fluctuations than the inhibitor-bound BRD4. The RMSD of the H1B-BRD4 is distributed at 1.69 and 2.30 Å, while that of the JQ1-BRD4 is populated at 1.69 and 1.30 Å and that of the TVU-BRD4 is located at 1.72 and 2.42 Å (Figure 5C). The RMSD of H1B-BRD9 is situated at 1.70 and 2.61 Å, while that of the JQ1-BRD9 is distributed at two bigger peak values of 2.09 and 3.07 Å and that of the TVU-BRD4 is populated at 1.60 and 2.31 Å (Figure 5D). Based on these results, the presence of three inhibitors produces a more evident influence on the structural stability of BRD9 than BRD4.

**Figure 6:**
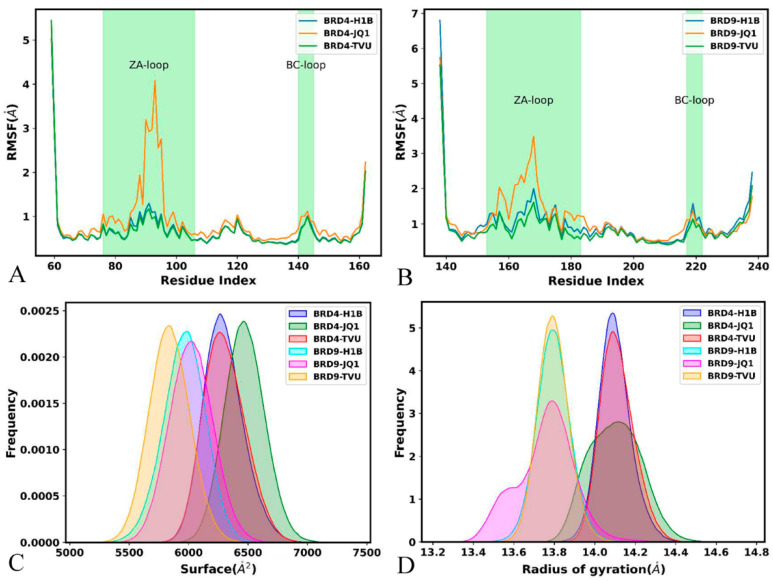
RMSFs of the Cα atoms from BRD4 and BRD9, molecular surface area and radius of gyration: (**A**) the RMSFs of BRD4, (**B**) the RMSFs of BRD9, (**C**) the frequency distribution of molecular surface area and (**D**) the frequency distribution for radius of gyrations.

The root-mean-square fluctuations (RMSFs) of BRD4 and BRD9 were estimated by using the coordinates of the Cα atoms (Figure 6A,B). It is found that the ZA-loop and BC-loop of BRD4 and BRD9 are highly flexible, especially for the ZA-loop. Compared to H1B and TVU, binding of JQ1 increases the structural flexibility of the ZA-loop from BRD4 and BRD9, but the structural flexibility of the ZA-loop in the JQ1-bound BRD4 is stronger than that in the JQ1-bound BRD9 (Figure 6A,B). Additionally, it is found that the difference in the structural flexibility of the other structural regions is tiny (Figure 6A,B). These results suggest that the electrostatic property of JQ1 highly affect the structural flexibility of the ZA-loop in two BRD proteins.

Molecular surface areas (MSAs) were estimated to examine the impacts of inhibitor associations on the solvent accessible extents of BRD4 and BRD9. The function of MSAs as the simulation time was displayed in Figure S6. The MSAs of the inhibitor-bound BRD4 are located at a range from 5651 to 7378 Å^2^, while those of the inhibitor-bound BRD9 fluctuate from 5118 to 6862 Å^2^. The MSAs of the H1B-, JQ1- and TVU-bound BRD4 are distributed at 6266, 6467 and 6265 Å^2^ (Figure 6C), respectively, while the MSAs of the H1B-, JQ1- and TVU-bound BRD9 are situated at the peak values of 5993, 6022 and 5831 Å^2^ (Figure 6C), individually. The results indicate that the contact extent of BRD4 with solvent is higher than BRD9, which shows the difference in hydrophily between BRD4 and BRD9.

The Radius of gyrations (RGs) were computed to check the effect of inhibitor binding on the structural compacts of the BRD4 and BRD9. The time course of RGs for BRD4 and BRD9 was exhibited at Figure S7. The RGs of the inhibitor-bound BRD4 fall into a range of between 13.71 and 14.74 Å, while those of the inhibitor-bound BRD9 fluctuate from 13.25 to 14.46 Å. The RGs of the H1B-, JQ1- and TVU-bound BRD4 are situated at ~14.08 Å (Figure 6D), and the RGs of the H1B-, JQ1- and TVU-bound BRD9 are populated at the peak values of ~13.79 Å (Figure 6D), indicating that the structural compact extent of the inhibitor-bound BRD4 is slightly lower than the inhibitor-bound BRD9. The aforementioned analyses suggested that the electrostatic property of JQ1 exerts certain influences on the structural stability of BRD4 and BRD9.

### 2.4. MM-GBSA Calculations

The binding affinity of H1B, JQ1 and TVU to BRD4/BRD9 was estimated through the MMPBSA.py in Amber to understand their binding strength, and the results are shown in Table 1 and Table 2. In our current calculation, 400 snapshots extracted from the equilibrated MD trajectories were adopted. It is found that the rank of our calculated results is of good consistence with that determined by the known experimental data. More interestingly, the binding ability of H1B, JQ1 and TVU to BRD9 is stronger than that of those to BRD4.

According to Table 1 and Table 2, the EIs ($∆E_{ele}$) of H1B and TVU with BRD4 are −29.97 and −34.45 kcal/mol, respectively, while that of these two inhibitors with BRD9 are −43.82 and −39.64 kcal/mol, individually, which is favorable for the binding of H1B and TVU to BRD4/BRD9. Additionally, the EIs of H1B and TVU with BRD9 are stronger than that with BRD4. The EI of JQ1 with BRD4 is −22.91 kcal/mol, which is favorable for the JQ1-BRD4 binding, while that of JQ1 with BRD9 is 15.43 kcal/mol, impairing the JQ1-BRD9 binding.

In comparison to the EIs, the polar solvation free energies ($∆G_{gb}$) of the inhibitor-BRD4/BRD9 complexes provide opposite contributions for inhibitor binding. On the whole, the sum ($∆G_{pol}$) of the $∆E_{ele}$ and $∆G_{gb}$ for six current complexes are unfavorable for the binding of inhibitors. It is noted that the VDWIs ($∆E_{vdW}$) of inhibitors with BRD4 and BRD9 are advantageous for the associations of inhibitors. The VDWIs of H1B, JQ1 and TVU with BRD9 are strengthened by 6.96, 11.86 and 5.67 kcal/mol compared to that of H1B, JQ1 and TVU with BRD4, respectively (Table 1 and Table 2). The non-polar solvation free energies ($∆G_{surf}$) also provide advantageous contributions to the associations of inhibitors, and the $∆G_{surf}$ of the H1B-, JQ1- and TVU-BRD9 complexes are individually enhanced by 0.59, 1.08 and 0.36 kcal/mol relative to that of the $∆G_{surf}$ of the H1B-, JQ1- and TVU-BRD4 complexes (Table 1 and Table 2). The entropy contributions (−T∆S) of the H1B-, JQ1- and TVU-BRD4 complexes are 18.65, 15.01 and 19.34 kcal/mol, separately, while that of the H1B-, JQ1- and TVU-BRD9 complexes are 25.11, 20.24 and 22.58 kcal/mol, individually (Table 1 and Table 2), which greatly impairs the binding of inhibitors to BRD4/BRD9. By referencing the inhibitor-BRD4 complexes, the −T∆S of the H1B-, JQ1- and TVU-BRD9 complexes are increased by 6.46, 5.23 and 3.24 kcal/mol, respectively, indicating that the entropy contributions produce a greater reduction in the binding ability of inhibitors to BRD9 than BRD4. The sum of two favorable forces ($∆E_{vdW}\;and\;∆G_{surf}$) of the H1B-, JQ1- and TVU-BRD9 complexes are strengthened 7.55, 12.94 and 6.03 kcal/mol compared to that of the H1B-, JQ1- and TVU-BRD9 complexes, individually. On the whole, the binding ability of H1B-, JQ1- and TVU to BRD9 is enhanced by 5.29, 5.56 and 6.32 kcal/mol relative to their binding abilities to BRD4. It is concluded that two favorable forces $∆E_{vdW}\;and\;∆G_{surf}$ play important roles in the increase in binding ability for H1B-, JQ1- and TVU to BRD9 in comparison to BRD4. Thus, VDWIs and non-polar solvation free energies should be given special attention in the design of highly selective inhibitors in regard to BRD4 over BRD9.

### 2.5. Interaction Network of Inhibitors with BRD9 and BRD4

**Figure 7:**
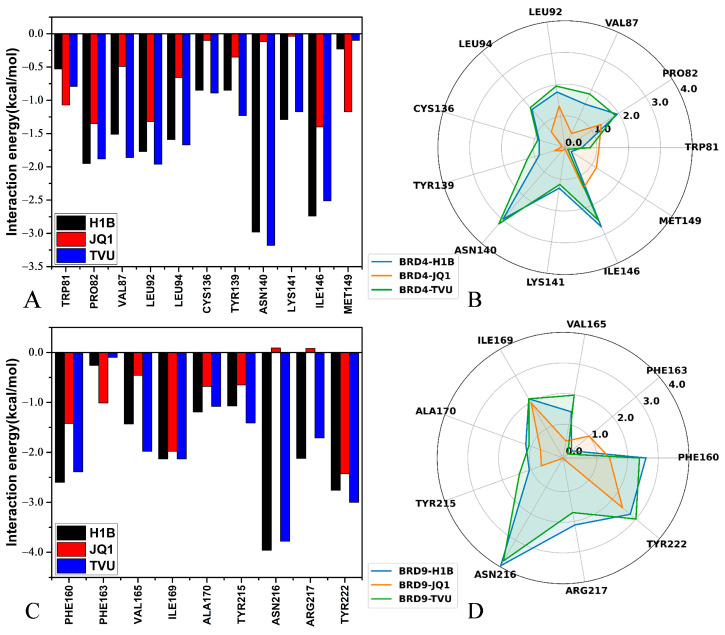
Key residues playing important roles in binding of inhibitors to BRD4 and BRD9: (**A**) BRD4, (**B**) the radar representation of inhibitor−residue interactions in BRD4, (**C**) BRD9 and (**D**) the radar representation of inhibitor−residue interactions in BRD9.

**Figure 8:**
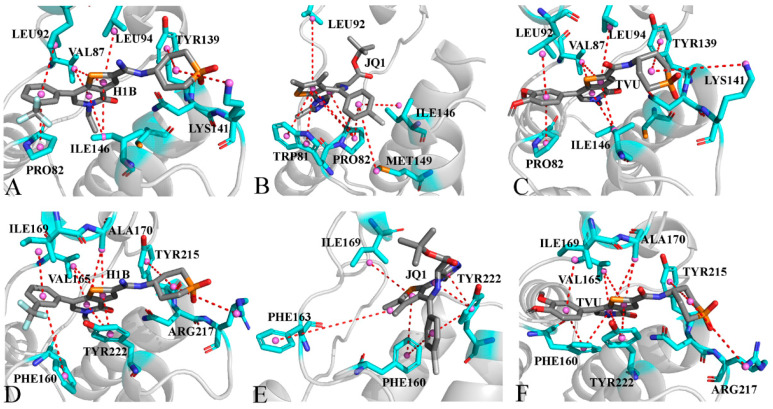
Hydrophobic interactions of inhibitors with residues in BRD4 and BRD9: (**A**) H1B-BRD4, (**B**) JQ1-BRD4, (**C**) TVU-BRD4, (**D**) H1B-BRD9, (**E**) JQ1-BRD9 and (**F**) TVU-BRD9.

**Figure 9:**
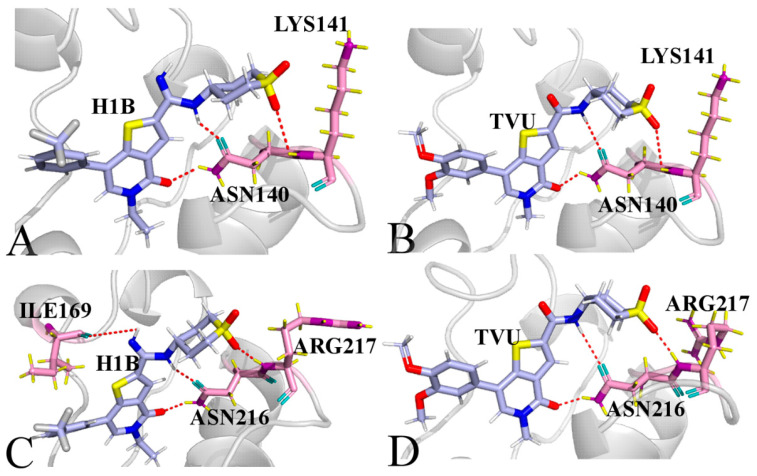
Hydrogen bonding interactions of inhibitors with BRD4 and BRD9: (**A**) H1B-BRD4, (**B**) TVU-BRRD4, (**C**) H1B-BRD9 and (**D**) TVU-BRD9.

To understand the contributions of separate residues to inhibitor-BRD4/BRD9 binding, inhibitor−residue interactions were estimated by means of a residue-based free energy decomposition method, and the results are provided in Figure 7 and Figure S8. The hydrogen bonding interactions (HBIs) of H1B, JQ1 and TVU with BRD4 and BRD9 were examined through the CPPTRAJ program in Amber, and the data is listed in Table 3 and Table 4. The geometric information on hydrophobic interactions and HBIs were displayed in Figure 8 and Figure 9. It was noted that three inhibitors (H1B, JQ1 and TVU) produce binding differences with separate residues in BRD4 and BRD9.

For the H1B-BRD4 complex, H1B produces interactions stronger than 0.8 kcal/mol with six residues, including PRO82, VAL87, LEU92, LEU94, TYR139 LYS141 and ILE146 (Figure 7A,B and Figure S8A). According to Figure 8A, the hydrophobic groups of PRO82, VAL87, LEU92, LEU94 and ILE146 are located near the hydrophobic ring of H1B. Thus, PRO82 structurally forms the π-π interaction of −1.95 kcal/mol with H1B. The alkyls of the residues VAL87, LEU92, LEU94 and ILE146 yield the CH-π interactions and their corresponding interaction energies are −1.51, −1.77, −1.59 and −2.74 kcal/mol, respectively (Figure 7A,B and Figure S8A). Structurally, the phenyl ring of TYR139 is next to the hydrophobic group of H1B, which leads to a π-π interaction of −0.85 kcal/mol. In addition, H1B also forms two HBIs with ASN140, and their occupancy is higher than 87.02%, indicating that these two hydrogen bonds are stable through the entire MD simulations (Table 3 and Figure 9A). A hydrogen bond with an occupancy of 61.73% appears between H1B and LYS141 (Table 3 and Figure 9A), implying that this hydrogen bond is also stable. As for the H1B-BRD9, eight residues generate interactions stronger than 0.8 kcal/mol, involving PHE160, VAL165, ILE169, ALA170, TYR215, ASN216, ARG217 and TYR222 (Figure 7C,D and Figure S7B). The phenyl groups of PHE160, TYR215 and TYR222 are adjacent to the hydrophobic ring of H1B, and, as a result, they are easy to yield to the π-π interactions (Figure 8D). Thus, PHE160, TYR215 and TYR222 contribute the interaction energies of −2.6, −1.07 and −2.76 kcal/mol to the H1B-BRD9 binding (Figure 7C,D and Figure S7B). The alkyls of VAL165, ILE169 and ALA170 are close to the hydrophobic ring of H1B, which leads to the formation of the CH-π interactions between them. Additionally, ILE169 forms a hydrogen bond with H1B, and its occupancy is 54.8% (Table 4 and Figure 9D). Hence, VAL165, ILE169 and ALA170 provide energy contributions of −1.43, −2.13 and −1.19 kcal/mol for the H1B-BRD9 association, respectively (Figure 7C,D and Figure S8B). The positive charge group of ARG217 is situated near the hydrophobic ring of H1B, and they form a cation-π interaction (Figure 8D). Meanwhile, ARG217 also forms a hydrogen bond with an occupancy of 96.82% with H1B (Table 4 and Figure 9D), and therefore ARG217 contributes an interaction energy of −2.12 kcal/mol to the H1B-BRD9 association. Furthermore, ASN216 form two hydrogen bonds with an occupancy of 99.16 and 99.64% with H1B (Table 4 and Figure 9D); as a result, ASN216 yields an interaction of −3.96 kcal/mol with H1B (Figure 7C,D and Figure S8B). Compared to BRD4, H1B produces more interactions with BRD9, which leads to a tighter binding to BRD9 than BRD4.

As for the JQ1-BRD4 complex, the interaction of JQ1 with five residues in BRD4 is stronger than 0.8 kcal/mol, and these five residues include TRP81, PRO82, LEU92, ILE146 and MET149 (Figure 7A,B and Figure S8B). The hydrophobic sidechains of TRP81 and PRO82 are located near the hydrophobic ring of JQ1, and they tend to form an π-π interaction (Figure 8B) that, respectively provides energy contributions of −1.07 and −1.35 kcal/mol for the JQ1-BRD4 binding. It is also observed that the hydrophobic sidechains of LEU92, ILE146 and MET149 are next to the hydrophobic ring of JQ1 (Figure 8B), and thus, structurally, they make it easy to produce the CH-π interactions. As a result, LEU92, ILE146 and MET149 individually contribute the interaction energy of −1.32, 1.4 and −1.17 kcal/mol to the JQ1-BRD4 associations (Figure 7A,B). With respect to the JQ1-BRD9 complex, four residues (PHE160, PHE163, ILE169 and TYR222) are involved in the interactions stronger than 0.8 kcal/mol with JQ1 (Figure 7C,D and Figure S8E). The interaction energies of PHE160, PHE163 and TYR222 with JQ1 are −1.42, −1.01 and −2.43 kcal/mol, which structurally agrees with the π-π interactions between JQ1 and these three residues (Figure 8E). The interaction strength of ILE169 with JQ1 is −1.98 kcal/mol, which structurally matches the CH-π interaction between JQ1 and ILE169 (Figure 8E).

Concerning the TVU-BRD4 complex, nine residues yield interactions stronger than 0.8 kcal/mol with TVU, and these eight residues include PRO82, VAL87, LEU92, LEU94, CYS136, TYR139, ASN140, LYS141 and ILE146 (Figure 7A,B and Figure S8C). According to Figure 8E, the alkyl groups of VAL87, LEU92, LEU94 and ILE146 are close to the hydrophobic ring of TVU, and thus the CH-π interactions are easy to form between them, and they provide energy contributions of −1.86, −1.96, −1.67 and −2.51 kcal/mol for the TVU-BRD4 binding (Figure 7A,B, Figure 8C and Figure S8C). The hydrophobic sidechains of PRO82 and TYR139 are situated near the hydrophobic rings of TVU, which structurally produces the π-π interactions (Figure 8C), and these two π-π interactions contribute the interaction energies of −1.88 and −1.23 kcal/mol to the TVU-BRD4 associations (Figure 7A,B and Figure S8C). The positive charge group of LYS141 structurally forms the cation-π interactions with the hydrophobic ring of TVU (Figure 8C). Meanwhile, LYS141 also forms a hydrogen bond with TVU, and its occupancy is 65.18% (Table 3 and Figure 9B); hence LYS141 provides an energy contribution of −1.17 kcal/mol to the TVU-BRD4 association (Figure 7A,B). In addition, ASN140 yields two HBIs with TVU and their occupancy is higher than 78.73% (Table 3 and Figure 9B), which leads to an interaction energy of −3.18 kcal/mol with TUV (Figure 7A,B). With regard to the TVU-BRD9 complex, TVU produces interactions stronger than 0.8 kcal/mol with eight residues, including PHE160, VAL165, ILE169, ALA170, TYR215, ASN216, ARG217 and TYR222 (Figure 7C,D and Figure S8F). The alkyls of VAL165, ILE169 and ALA170 are adjacent to the hydrophobic ring of TVU, and it is easy to form the CH-π interactions between them (Figure 8F). As a result, VAL165, ILE169 and ALA170, respectively, contribute the interaction energies of −1.98, −2.13 and −1.08 kcal/mol to the TVU-BRD9 associations (Figure 7C,D and Figure S8F). The hydrophobic rings of PHE160, TYR215 and TYR222 are next to that of TVU, which leads to the π-π interactions between them (Figure 8F). PHE160, TYR215 and TYR222 provide energy contributions of −2.39, −1.41 and −3.00 kcal/mol for the binding of TVU to BRD9 (Figure 7C,D and Figure S8F). According to Figure 8F, the positive charged group of ARG217 is located near the hydrophobic ring of TVU, which induces a cation-π interaction between them. In addition, ARG217 forms a hydrogen bond with TVU, and its occupancy is 96.72% (Table 4 and Figure 9D). In total, ARG217 contributes an energy contribution of −1.71 kcal/mol to the TVU-BRD9 association (Figure 7C,D). ASN216 generates two HBIs with TVU, and their occupancy is higher than 69.30%; this residue gives an energy contribution of −3.78 kcal/mol for binding of TVU to BRD9 (Table 4 and Figure 9D).

Based on the aforementioned analyses, it is found that residues (PRO82, PHE160), (LEU92, ILE169) and (ILE146, TYR222) in (BRD4, BRD9) play important roles in the binding of H1B, JQ1 and TVU to BRD4/BRD9. The CH-π, π-π, cation-π interactions and HBIs are responsible for the primary forces for the binding of inhibitors to BRD4 and BRD9; therefore, these common factors involved in inhibitor−residue interactions should be given special attention in future drug designs in regard to the BRD family.

## 3. Materials and Methods

### 3.1. Preparation of Simulation Systems

The initial atom coordinates of the JQ1-BRD4, H1B-BRD9 and TVU-BRD9 complexes were taken from the protein data bank (PDB), and they correspond to PDB entry 3MXF [36], 6V1B [24] and 4UIU [4], respectively. Due to differences in the residue sequences in BRD4 and BRD9, residues K59-E162 in BRD4 and residues E138-S239 were adopted to construct our simulation systems. Because of unavailable crystal structure of the JQ1-BRD9 complex in PDB, the JQ1-BRD9 structure was obtained by deleting BRD4 and H1B from the superimposed structures of 3MXF with 6V1B. The H1B-BRD4 and TVU-BRD4 structures were produced by removing BRD9 and JQ1 from the aligned structures of 3MXF with 6V1B and 4UIU. Apart from the inhibitors, the other ligands were cut from the initial model. The protonated states of residues from BRD4 and BRD9 were checked by means of the program H++ 3.0 [71], and rational protonation states were given to each residue of BRD4 and BRD9. The missing hydrogen atoms in the crystal structures were connected to their corresponding heavy atoms by using the Leap module in Amber 22 [72,73]. The ff19SB force field [74] was used to derive the parameters of BRD4 and BRD9. The structures of three inhibitors (JQ1, H1B and TVU) were optimized at a semi-empirical AM1 level, and, subsequently, the BCC charges [75,76] were assigned to each atom of three inhibitors by using the Antechamber module in Amber [77]. The general Amber force field (GAFF2) [78,79] was utilized to get the force field parameters of JQ1, H1B and TVU. The JQ1-, H1B- and TVU-bound BRD4/BRD9 were solved at an octahedral periodic box of water with a buffer of 10.0 Å to embody the solvent environment, while the force field parameters of water molecules were derived from the TIP3P mode [80]. The appropriate number of sodium ions (Na^+^) and chloride ions (Cl^−^) were placed in the water box in 0.15 M NaCl salt concentration to form the neutral simulation systems and the parameters of the Na^+^ and Cl^−^ ions were extracted from the studies of Joung et al. [81,82].

### 3.2. Multiple Independent Molecular Dynamics

Initialization of six BRD4 or BRD9-related systems possibly results in high energy contacts and orientations between atoms of the entire systems, which may disturb the stability of system simulations. In order to overcome this issue, all of six BRD4- or BRD9-related systems were subjected to a 5000-cycle steepest descent minimization followed by a 10,000-cycle conjugate gradient minimization. The temperature of six optimized systems was softly increased from 0 to 300 K within 2 ns in the canonical ensemble (NVT), and, in this process, all non-hydrogen atoms of the BRD4- or BRD9-related systems were restrained in a weak harmonic restriction of 2 kcal·mol^−1^·Å^2^. Then, a 3-ns equilibrium process was conducted on six current systems at 300 K under the isothermal–isobaric ensemble (NPT). Then, the 15-ns NPT simulation was implemented to keep the density of the system at 1.01 g/cm^3^. Subsequently, three 300-ns independent MD simulations were separately performed on six current systems at the NVT with periodic boundary conditions and the particle mesh Ewald method (PME). In each independent MD simulation, the initial atomic velocities were randomly assigned with the Maxwell distribution. To facilitate the post-processing analysis, three independent MD trajectories were joined into a single MD trajectory (SMT). Through all MD simulations, the chemical bonds linking with hydrogen atoms were restricted by adopting the SHAKE algorithm [83]. The Langevin dynamics with a collision frequency of 2.0 ps^−1^ was executed to control the temperature of six BRD4- or BRD9-related systems [84]. The particle mesh Ewald (PME) method [85] cooperating with an appropriate cutoff value of 12 Å was aided to perform calculations of electrostatic interactions (EIs), and this cutoff was also utilized for treatment of the van der Waals interactions (VDWIs). The program pmemd.cuda [86,87] inlayed in Amber 22 was used to run cMD simulations.

### 3.3. Deep Learning

To probe impacts of inhibitor binding on internal structures of BRD4 and BRD9, DL was applied to identify the differences in residue contacts. The residue contact map in each MIMD trajectory snapshot of BRD4 and BRD9 was calculated by utilizing a Python packages MDTraj and contact map explorer. A contact definition of ≤4.5 Å between any Cα atoms of two proteins was employed. The derived 104 × 104 (102 × 102 for BRD9) residue contacts were transformed into the 104 × 104 (102 × 102 for BRD9) pixel grayscale images for the analysis by a two-dimensional (2D) convolutional neural network (CNN). In total, 120,000 images were produced for each BRD4-related or BRD9-related system, 80% of which were randomly selected for training, while the rest were used for validation. In this work, the 2D-CNN was built by using the PyTorch package (https://pytorch.org/). This CNN model was mainly composed of 2 convolutional layers of 1 × 1 kernel size, with 16, 32, filters, individually, followed by three fully connected layers, the first two of which included 512 and 128 filters with a dropout rate of 0.5 each. The final fully connected layer was the classification layer for the inhibitor-bound BRD4 or BRD9. Excluding the classification layers, the “ReLu” activation function was utilized in all layers of the 2D-CNN, in which the “softmax” activation function was adopted. A maximum pooling layer with 2 × 2 kernel size was added after each convolutional layer. Lastly, the backpropagation by vanilla gradient-based pixel attribution was employed to estimate the attention map of residue contact gradients so as to discriminate the function difference of BRD4 or BRD9 induced by inhibitor binding, in which the residue contact map was reflected using the most populated structural cluster of each BRD4- or BRD9-related system. We primarily referenced the work of Miao’s group and rewrote the program using PyTorch.

### 3.4. MM-GBSA Calculations

It is well known that binding free energy is regarded as an indicator to understanding binding strength of ligands to targets. The enthalpy changes (∆*H*) and entropy changes (−*T*∆*S*) are two important factors accompanying the associations of ligands. In this study, binding free energies of H1B, JQ1 and TVU to BRD4/BRD9 were calculated based on the following equation to evaluate binding difference of inhibitors to BRD4 and BRD9

∆*G* = ∆*H* − *T*∆*S* (1)

By now, the molecular mechanics Poisson−Boltzmann surface area (MM-PBSA) and MM-GBSA are two efficient methods for quickly computing binding-free energies [88,89]. Sun et al. compared the performance of MM-PBSA and MM-GBSA in predictions of binding free energies, and their works uncovered that MM-GBSA obtained a more favorable results than MM-PBSA [90,91]. As for MM-GBSA calculations, ∆*H* is further divided into two separate components that are included in Equation (2)

(2) $$\begin{array}{c} ∆H=∆G_{pol}+∆G_{hydro}\\ =∆E_{ele}+∆E_{vdW}+∆G_{gb}+∆G_{surf} \end{array}$$

where $∆G_{pol}=∆E_{ele}+∆G_{gb}$ is polar interactions between inhibitors and proteins, while $∆G_{hydro}=∆E_{vdW}+∆G_{surf}$ indicates hydrophobic interactions of inhibitors with proteins. In details, EIs ($∆E_{ele})$ and VDWIs ($∆E_{vdW})$ can be estimated with molecular mechanics and the ff19SB force field. Polar solvation free energies ($∆G_{gb})$ are estimated through the GB model proposed by Onufriev et al. [92]. Non-polar solvation free energies ($∆G_{surf}$) are calculated according to the following empirical Equation (3):

(3) $$∆G_{surf}=\gamma \times ∆SASA+\beta$$

where the term ∆SASA implies the difference in the solvent accessible surface area caused by the associations of inhibitors. The entropy changes −T∆S are calculated by means of the MMPBSA.py program in Amber [93]. The two parameters γ and β are set as 0.0072 kcal·mol·Å^−2^ and 0.0 kcal·mol^−1^, separately [94].

### 3.5. Principal Component Analysis

PCA is an important method for deciphering the conformational dynamics of proteins. In this study, PCA was achieved by performing diagonalization on the covariance matrix built using the coordinates of the atoms Cα of BRD4 and BRD9 recoded in the SMT based on Equation (4)

(4) $$C=<(q_{i}-<q_{i}>){\left(q_{j}-<q_{j}>\right)}^{T}>$$

In which $q_{i}$ and $q_{j}$ are the Cartesian coordinates of the *i*th and *j*th Cα atoms in BRD4 and BRD9, respectively, and the terms $<q_{i}>$ and $<q_{j}>$ are their averaged positions over conformational ensembles resulting from MIMD simulations. The eigenvector and the eigenvalue generated by diagonalization, respectively, characterize concerted movement of the structural domains and the fluctuation amplitude along an eigenvector. In this work, the PCA was realized by using the CPPTRAJ program in Amber.

## 4. Conclusions

BRD4 and BRD9 have been identified critical targets of drug development for treatment of various human diseases. Studying of the atomic-level binding mechanism of inhibitors to BRD4 and BRD9 has proven to be significant. Three independent MD simulations, each for running 400 ns, were carried out to improve conformational samplings of BRD4 and BRD9, and the post-processing analysis based on MD trajectory indicates that the electrostatic property of inhibitors highly affects the structural flexibility of the ZA-loop of BRD4 and BRD9. The MD trajectory-based DL reveals that the ZA-loop and BC-loop play critical roles in the function of BRD4 and BRD9. The calculated binding free energies through the MM-GBSA method not only indicate that VDWIs are responsible primary forces in bindings of inhibitors to BRD4/BRD9, but also suggest that the binding abilities of H1B, JQ1 and TVU to BRD9 are stronger than BRD4. The results from PCA implies that the electrostatic property of JQ1 greatly alters the dynamics behavior of the ZA-loop in BRD4 and BRD9. The interaction network analysis reveals key residues in inhibitor binding and provides targeting sites of drug design in regard to BRD4 and BRD9.

## Figures and Tables

**Table 1:** Binding free energies of inhibitors to BRD4 obtained by MM-PBSA method.

Complex	H1B		JQ1		TVU	
	Average	Std	Average	Std	Average	Std
$∆E_{ele}$	−29.97	9.39	−22.91	15.93	−34.45	9.84
$∆E_{vdw}$	−41.78	3.38	−28.51	7.13	−44.36	2.92
$∆G_{gb}$	42.71	7.61	33.53	15.38	48.59	7.69
$∆G_{surf}$	−5.70	0.44	−3.50	0.90	−5.97	0.33
∆Gpola	12.74	12.09	10.62	22.14	14.14	12.49
−T∆S	18.65	5.52	15.01	6.13	19.34	6.15
∆Gbindb	−16.09	5.81	−6.38	6.54	−16.85	6.44
∆Gexpc			−10.00			

**Table 2:** Binding free energies of inhibitors to BRD9 obtained by MM-PBSA method.

Complex	H1B		JQ1		TVU	
	Average	Std	Average	Std	Average	Std
$∆E_{ele}$	−43.82	7.69	15.43	9.03	−39.46	4.91
$∆E_{vdw}$	−48.74	2.89	−40.37	4.25	−50.03	3.28
$∆G_{gb}$	52.35	6.15	−2.66	8.85	50.06	4.22
$∆G_{surf}$	−6.29	0.30	−4.58	0.41	−6.33	0.29
∆Gpola	3.62	6.80	12.77	12.65	10.60	6.48
−T∆S	25.11	4.35	20.24	3.75	22.58	4.15
∆Gbindb	−21.38	6.08	−11.94	5.55	−23.17	5.26
∆Gexpc	−10.16				−11.35	

Note: Standard errors are given in parentheses. ∆Gpola=∆Eele+∆Ggb; ∆Gbindb=∆Eele+∆Gpol+∆Evdw+∆Gsurf−T∆S; ∆Gexpc The experimental values were derived from the experimental Ki values in Reference using the equation ${∆G}_{exp}=-RTln{IC}_{50}$.

**Table 3:** The hydrogen bonds formed between key residues and inhibitors for BRD4.

Inhibitor	Donor	Acceptor	^a^ Distance(Å)	^a^ Angle(°)	^b^ Occupied(%)
H1B	ASN140:ND2-HD21	H1B:O33	3.10	158.21	87.02
	H1B:N37-H13	ASN:140:OD1	3.96	157.19	90.26
	LYS141:N-H	H1B:O49	2.96	160.86	61.73
TVU	ASN140:ND2-HD21	TVU:O2	2.94	158.70	99.62
	TVU:N1-H4	ASN140:OD1	3.20	152.23	78.73
	LYS141:N-H	TVU:O5	2.94	159.11	65.18

^a^ The hydrogen bonds are determined by the donor…acceptor atom distance of <3.5 Å and acceptor… H-donor angle of >120°. ^b^ Occupancy is used to evaluate the stability and strength of the hydrogen bond.

**Table 4:** The hydrogen bonds formed between key residues and inhibitors for BRD9.

Inhibitor	Donor	Acceptor	^a^ Distance(Å)	^a^ Angle(°)	^b^ Occupied(%)
H1B	ASN216:ND2-HD21	H1B:O33	2.96	155.69	99.16
	H1B:N37-H13	ASN216:OD1	2.93	160.29	99.64
	ARG217:N-H	H1B:O49	2.96	164.08	96.82
	H1B:N35-H12	ILE169:O	3.00	153.78	54.80
TVU	ASN216:ND2-HD21	TVU:O2	2.86	157.25	99.96
	TVU:N1-H10	ASN216:OD1	3.26	152.05	69.30
	ARG217:N-H	TVU:O5	2.96	160.72	96.72

^a^ The hydrogen bonds are determined by the donor…acceptor atom distance of <3.5 Å and acceptor… H-donor angle of >120°. ^b^ Occupancy is used to evaluate the stability and strength of the hydrogen bond.

## Data Availability

Data are contained within the article and Supplementary Materials.

## Supplementary Materials

The following supporting information can be downloaded at: https://www.mdpi.com/article/10.3390/molecules29081857/s1, Figure S1. Workflow for deep learning on MD trajectories; Figure S2. Learning curves of the training and validation datasets for inhibitor-bound BRD4 and BRD9; Figure S3. The function of eigenvalues over eigenvector indexes; Figure S4. Free energy profiles and representative structures of the inhibitor-bound BRD9; Figure S5. Concerted motions of BRD4 and BRD9 revealed by the first eigenvector from PCA; Figure S6. The time course of molecular surface area; Figure S7. The time evolution for radius of gyrations; Figure S8. Interactions of inhibitors with separate residues in BRD4 and BRD9.
