# Supplementary material for: Binding Mechanism of Inhibitors to BRD4 and BRD9 Decoded by Multiple Independent Molecular Dynamics Simulations and Deep Learning
Source: Molecules. 2024 Apr 19;29(8):1857. doi: 10.3390/molecules29081857 (PMC11054187; doi:10.3390/molecules29081857)
Supplement: Supplementary file 1 [file molecules-29-01857-s001.zip › molecules-2972360-supplementary.pdf]

Binding mechanism of inhibitors to BRD4 and BRD9 decoded by multiple independent molecular dynamics simulations and deep learning

Jian Wang[1] Wanchun Yang[1], Lu Zhao[1], Benzheng Wei[2] and Jianzhong Chen[1]

1 School of Science, Shandong Jiaotong University, Jinan, 250357, China

2 Center for Medical Artificial Intelligence, Shandong University of Traditional Chinese Medicine, Qingdao 266112, China

* Correspondence: wangjian_lxy@sdjtu.edu.cn (J.W.); chenjianzhong1970@163.com(J.C.)

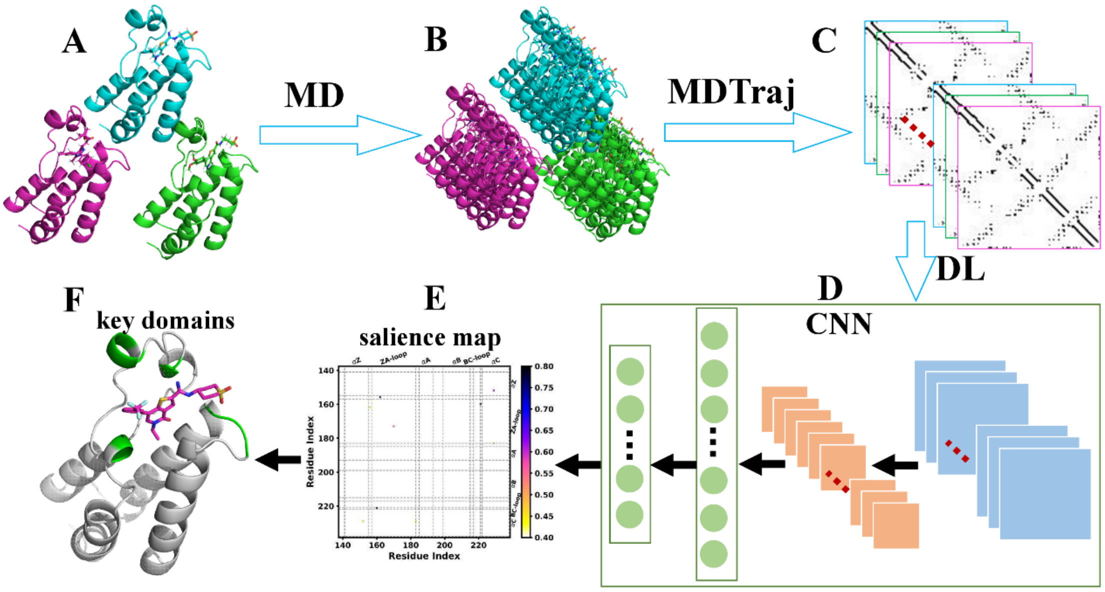

Figure S1. Workflow for deep learning on MD trajectories: (A) crystal structures, (B) conformations of inhibitor-BRD4/BRD9 complexes recorded MD trajectories, (C) images transformed from MD trajectories by using the MDTraj program, (D) convolutional neural network for classifications, (E) the salience maps of key residues detected by DL and (F) significant structural domains revealed by DL.

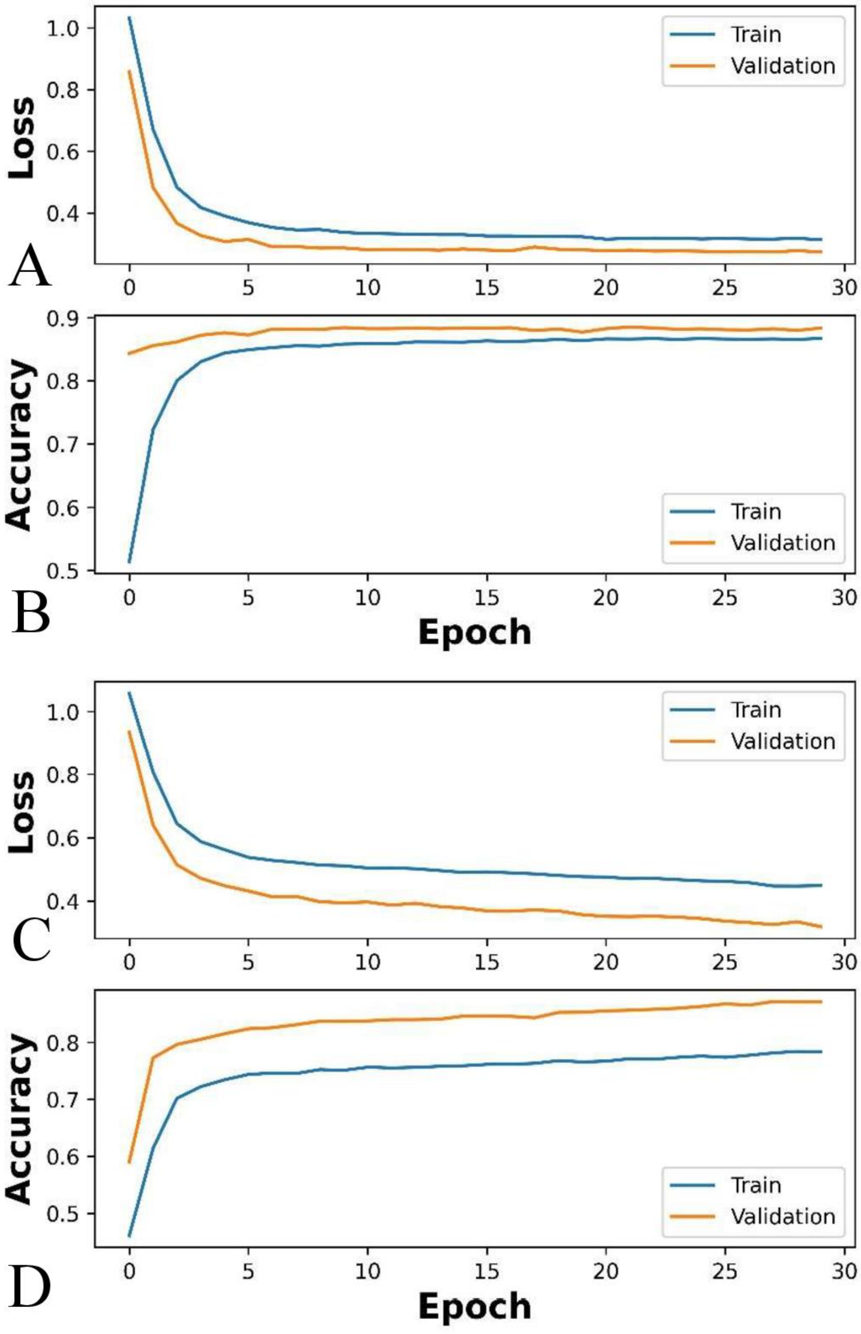

Figure S2. Learning curves of the training and validation datasets for inhibitor-bound BRD4 and BRD9: The metrics used here are loss and accuracy of inhibitor-bound BRD4 (A)-(B) and inhibitor-bound BRD9 (C)-(D).

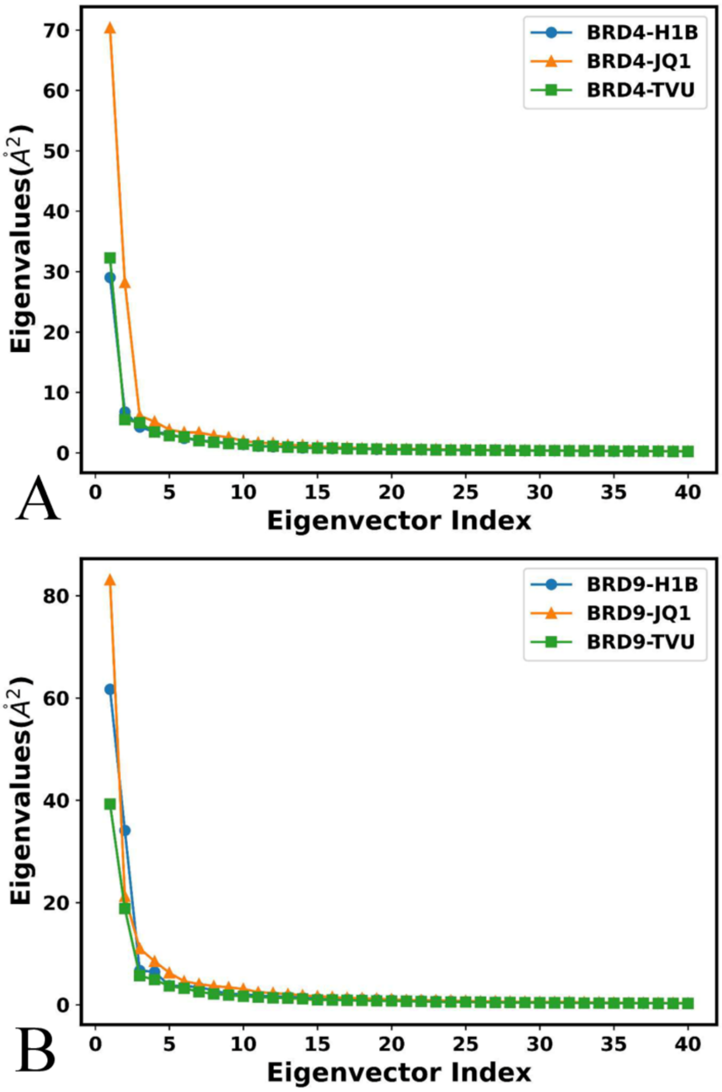

Figure S3. The function of eigenvalues over eigenvector indexes: (A) the inhibitor-bound BRD4 and (B) the inhibitor-bound BRD9.

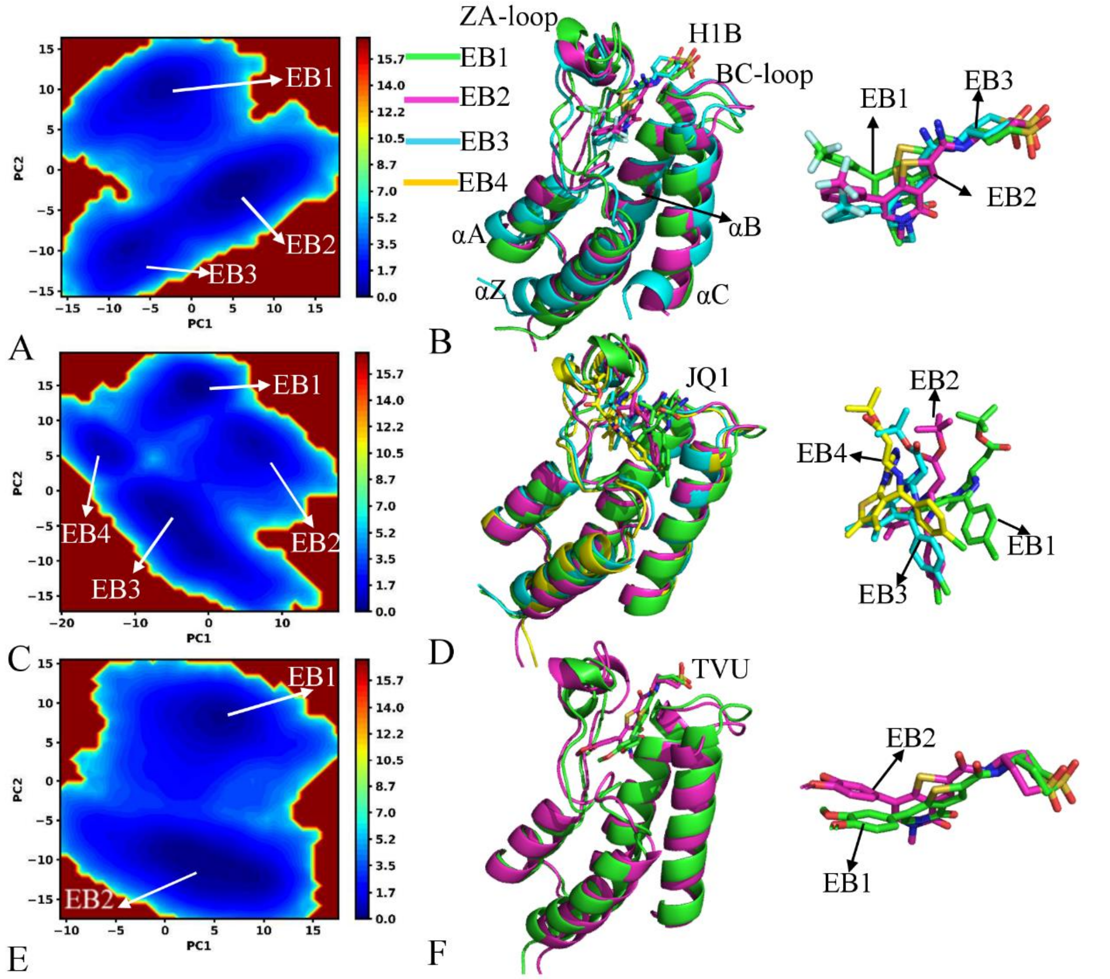

Figure S4. Free energy profiles and representative structures of the inhibitor-bound BRD9: (A), (C) and (E) corresponding to FELs of the H1B-, JQ1- and TVU-bound BRD9, respectively, and (B), (D) and (F) indicating superimposition of representative structures for BRD9 and three inhibitors trapped in different EBs. The free energy is scaled in kcal/mol and BRD9 is shown in cartoon modes.

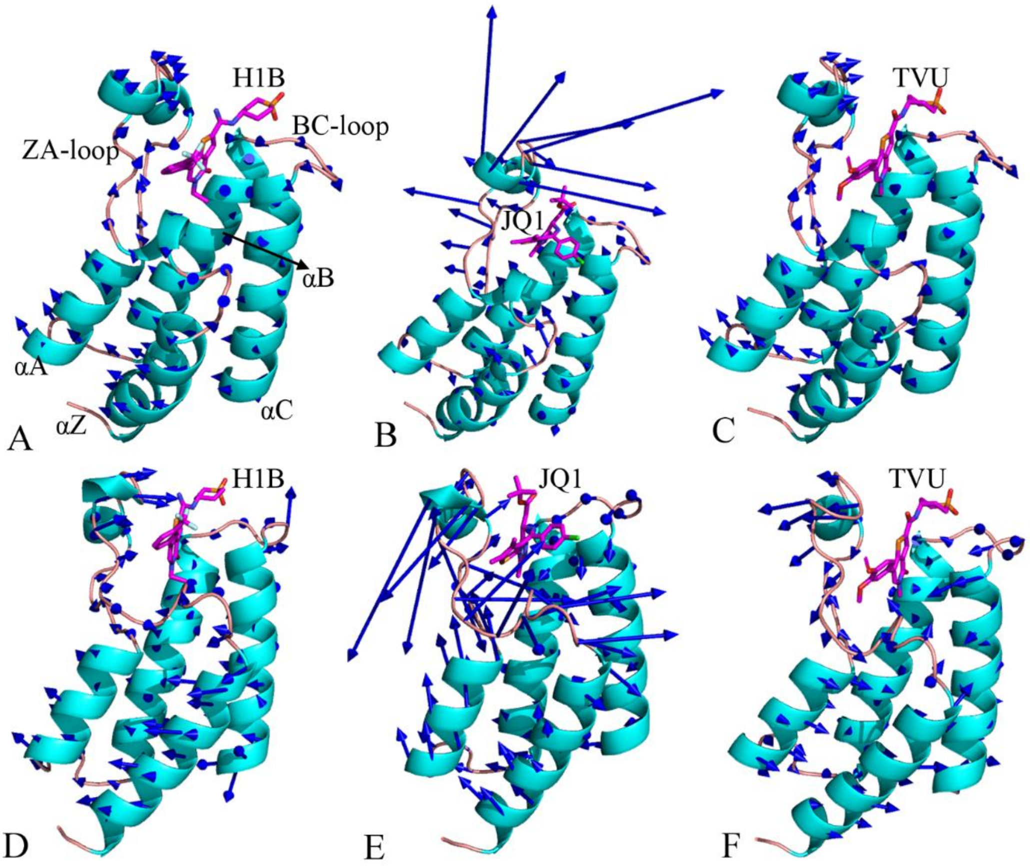

Figure S5. Concerted motions of BRD4 and BRD9 revealed by the first eigenvector from PCA: (A) the H1B-BRD4, (B) the JQ1-BRD4, (C) the TVU-BRD4, (D) the H1B-BRD9, (E) the JQ1-BRD9 and (F) the TVU-BRD9. In this figure, BRD4 and BRD9 were shown in cartoon modes and three inhibitors were displayed in stick modes.

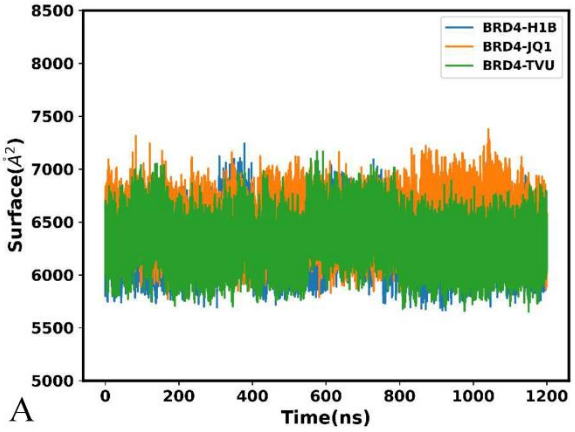

A

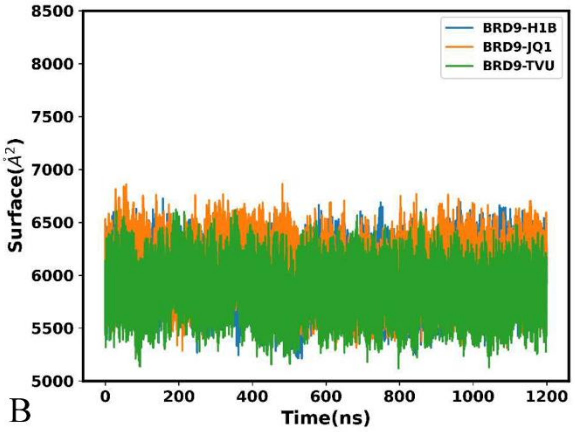

B

Figure S6. The time course of molecular surface area: (A) the inhibitor-bound BRD4 and (B) the inhibitor-bound BRD9.

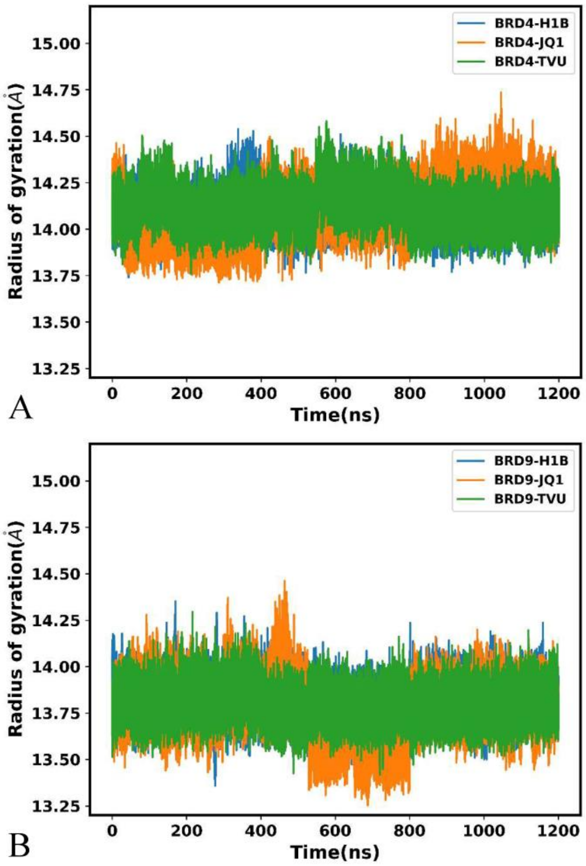

Figure S7. The time evolution for radius of gyrations: (A) the inhibitor-bound BRD4 and (B) the inhibitor-bound BRD9.

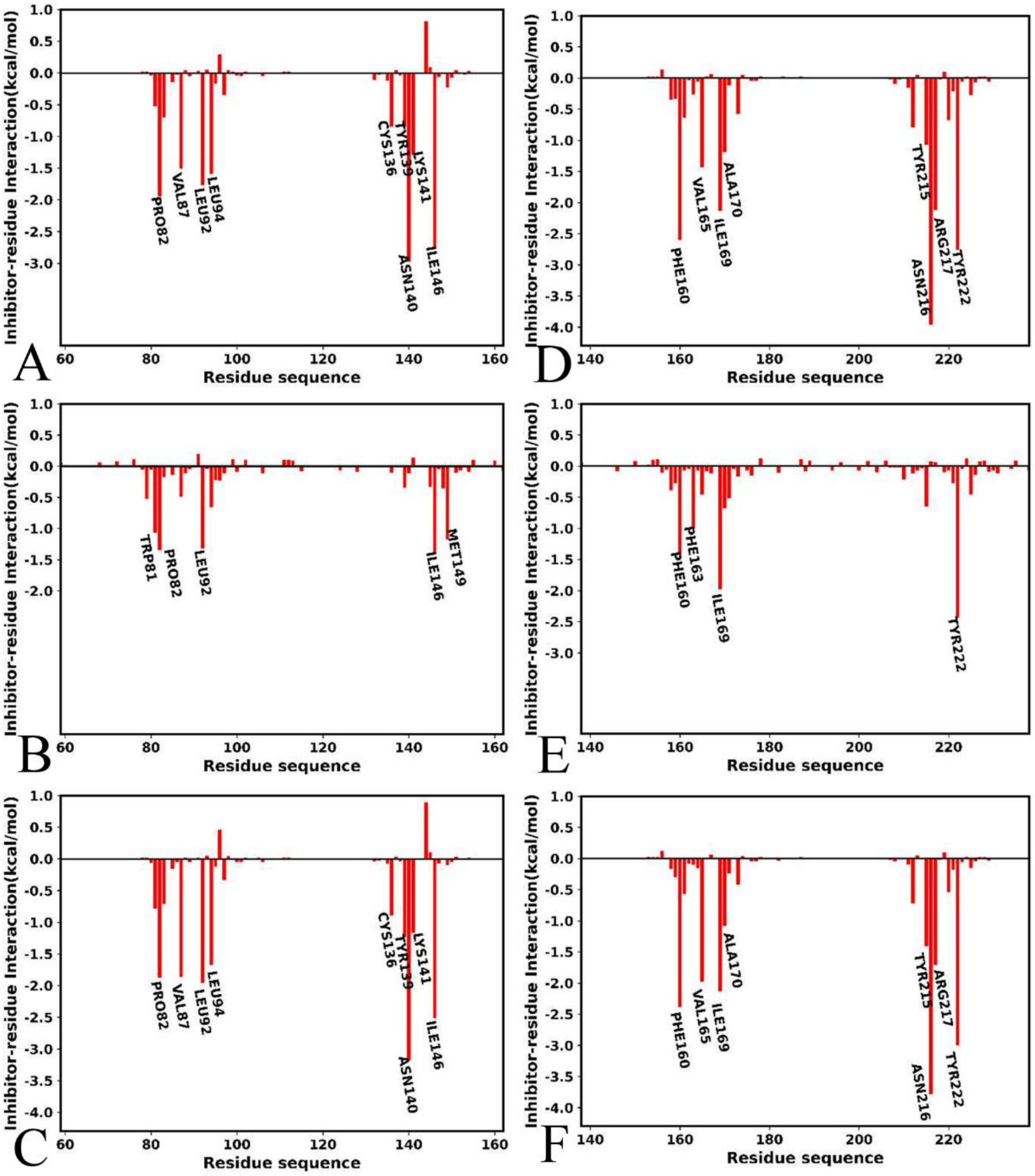

Figure S8. Interactions of inhibitors with separate residues in BRD4 and BRD9: (A) H1B-BRD4, (B) JQ1-BRD4, (C) TVU-BRD4, (D) H1B-BRD9, (E) JQ1-BRD9 and (F) TVU-BRD9.
